# Horizontally transferred genes cluster spatially and metabolically

**DOI:** 10.1186/s13062-015-0102-5

**Published:** 2015-12-21

**Authors:** Alexander Dilthey, Martin J. Lercher

**Affiliations:** Institute for Computer Science, Heinrich Heine University, 40225 Düsseldorf, Germany; Present address: The Wellcome Trust Centre for Human Genetics, University of Oxford, Oxford, UK

**Keywords:** Horizontal gene transfer, Lateral gene transfer, Co-transfer, Benchmark, Spatial clustering, Metabolic clustering, Flux balance analysis

## Abstract

**Background:**

Genomic uptake of DNA by prokaryotes often encompasses more than a single gene. In many cases, several horizontally transferred genes may be acquired together. Accordingly, we expect that horizontally transferred genes cluster spatially in the genome more often than expected if transfers were independent. Further, genes that depend on each other functionally may be unlikely to have beneficial fitness effects when taken up individually by a foreign genome. Hence, we also expect the co-acquisition of functionally related genes, resulting in the clustering of horizontally transferred genes in functional networks.

**Results:**

Analysing spatial and metabolic clustering of recent horizontal (or lateral) gene transfers among 21 γ-proteobacteria, we confirm both predictions. When comparing two datasets of predicted transfers that differ in their expected false-positive rate, we find that the more stringent dataset shows a stronger enrichment of clustered pairs.

**Conclusions:**

The enrichment of interdependent metabolic genes among predicted transfers supports a biologically significant role of horizontally transferred genes in metabolic adaptation. Our results further suggest that spatial and metabolic clustering may be used as a benchmark for methods that predict recent horizontal gene transfers.

**Reviewers:**

This article was reviewed by Peter Gogarten in collaboration with Luiz Thiberio Rangel, and by Yuri Wolf.

## Open peer review

Reviewed by Peter Gogarten in collaboration with Luiz Thiberio Rangel and by Yuri Wolf. For the full reviews, please go to the Reviewers’ comments section.

## Background

Horizontal gene transfer (HGT, also termed lateral gene transfer) plays a dominant role in prokaryotic evolution and adaptation [1–4]. In the best studied prokaryote, *Escherichia coli* K12, at least 18 % of all genes [5] and more than one third of transporters [4] are thought to be the result of horizontal transfers. While protein families differ in their frequencies of successful transfers [6], most types of genes seem to be able to transfer horizontally [7].

Successful horizontal gene transfer requires two steps: (1) the physical transfer of a piece of genetic information, and (2) the subsequent fixation of this macro-mutation in a population. Physical transfers are mediated by a range of processes, including transformation, transduction, conjugation, gene transfer agents, and nanotubes [8–10]. These mechanisms are by no means restricted to the transfer of exactly one gene – a substantial fraction of physical transfers will include either an incomplete gene or genetic information from more than one gene (“co-transfer”) [11]. If two or more complete genes are transferred together, they may also be integrated into the host genome together. Thus, co-transferred genes will often be genomic neighbours, even if some chromosomal shuffling may occur after their insertion. That co-transfers occur is evident from the transfer of gene sets encoded on plasmids [8]. Co-transfers may also be responsible for the existence of “genomic islands” harbouring strain-specific genes [12]; however, these clusters may also correspond to regions in which horizontally transferred genes are integrated preferentially [8]. Despite anecdotal evidence for genomic clustering of horizontally transferred genes, it is presently unclear to what extent such clustering is found throughout bacterial genomes.

Following the physical transfer, the newly acquired genes can spread through a bacterial population, and may eventually become fixed. This fixation may occur simply by random drift, evidenced by recently acquired genes that do not appear to contribute to their host’s fitness [13, 14]. However, bacterial genomes tend to loose non-beneficial DNA sequences [15], and non-beneficial horizontally transferred genes are thus unlikely to be retained for long evolutionary periods. In contrast, genes that increase their host’s fitness will be aided in their fixation by positive selection, which is particularly effective in many bacterial species due to their large population sizes [16]. This implies that certain genes are more likely to transfer successfully than others: for example, proteins that act by themselves are more likely to provide immediate fitness increases, while an enzyme whose action requires the presence of another enzyme is unlikely to be fixed unless its interaction partner is already present in the host [6, 17]. If such a gene is not transferred by itself, but together with its interaction partner(s), chances for a successful integration are expected to rise. Such co-transfers are further facilitated by the organisation of interacting proteins into operons. This argument is related to the “selfish operon” hypothesis [18], which posits that the close genomic linkage of functionally related genes is favoured by natural selection because it allows their joint transfer to other genomes. However, while the co-transfer of interacting genes is indeed facilitated by their co-localization in an operon, this may not be related to the reason why the operon was formed in the first place [19, 20]. Below, we analyse individual genomes rather than whole populations, and it is thus possible that some of the observed genes are not fixed in the population; however, genes fixed in a population are more likely to be observed simply because they are present in any genome chosen for sequencing.

Thus, there are reasons to believe that at least part of the genes acquired horizontally by one genome are related to each other (i) in chromosomal positioning (spatial clustering) and/or (ii) through their association in functional networks. Below, we test the generality of these two predictions by comparing the spatial and metabolic clustering of horizontally transferred genes to that of randomly chosen genes across 21 closely related γ-proteobacteria.

While the extent of functional clustering has not been quantified before, its existence has been used to identify gene functions from genomic data. In phylogenetic profiling [21], the statistically significant co-occurrence of two genes across several genomes is used as evidence for their functional association, thus allowing the transfer of annotations between them.

## Results and discussion

To identify recent horizontal transfers, we used previously published sets of HGT candidates [22]. In this previous work, gene presence/absence data for orthologous gene families was projected onto the terminal nodes of a well-resolved phylogenetic tree representing vertical inheritance from an ancestral species to 21 extant γ-proteobacteria. Horizontal gene transfers were identified based on the most parsimonious explanation of gene presence/absence [4]. Of 42,677 examined genes, 2,020 (equal penalties for gene gains and losses, *terminal.pen1*; see [4, 22] for details) and 961 (higher penalties for gene gains than for losses, *terminal.pen2*) putative horizontal gene transfers were mapped to the terminal branches of the phylogeny.

Based on our null model of no association between HGT and the distance of genes along the chromosome, we would expect to find 540 and 145 pairs of genes that are genomic neighbours, respectively, in the two datasets. We actually observe 882 and 401, corresponding to a 1.6- and 2.8-fold enrichment, respectively. Both enrichment values are statistically highly significant (Table 1).

**Table 1:** Clustering scores (*CC*) for spatial clustering of horizontally transferred genes across all examined genomes. Two horizontally transferred (HGT) genes were considered genomic neighbours if they had at most 2 intervening genes between them

Result set	# HGT candidates	Expected pairs	Observed pairs	*CC*	*p*
terminal.pen1	2020	540.17	882	1.633	<0.001
terminal.pen2	961	144.88	401	2.768	<0.001

The *Escherichia coli* K12 genome contains 205 (*terminal.pen1*) and 85 (*terminal.pen2*) genes predicted to be the result of recent horizontal transfers. If metabolic interactions were independent of HGT status, we would expect these candidates to contain about 5 and 1 pairs of interacting genes, respectively. We actually observe 24 and 0, respectively (Table 2). For the first, larger set of HGT predictions, this corresponds to a 5-fold enrichment, which is statistically highly significant (*p* < 0.001). The second dataset, with a null expectation of 1 pair, is too small to assess statistical significance.

**Table 2:** Clustering scores (*CC*) for metabolic clustering of genes recently transferred horizontally into *E. coli* K12. Two horizontally transferred (HGT) genes were considered metabolic neighbours if they encode reactions that catalyse tightly correlated fluxes in the *E. coli* K12 metabolic network

Method	# HGT candidates	Expected pairs	Observed pairs	*CC*	*p*
terminal.pen1	205	4.68	24	5.128	<0.001
terminal.pen2	85	0.80	0	0.000	0.76

Our results show clear evidence for a clustering of horizontally transferred genes, both spatially and functionally. The observed clustering scores, quantifying the degree of departure from our null models of no association, range from 1.6 to 5.1.

This analysis is based on rather conservative assumptions: only chromosomally encoded genes are included, only the most recent (therefore most reliably inferred) transfer events are taken into account, and genes without orthologs among the examined genomes are discarded. Finally, our statistical model biases the inferred clustering scores downwards and the inferred *p* values upwards, as it does not account for the gaps between potential HGT candidates induced by excluded genes.

We have chosen a method of generalised parsimony for the detection of HGT events, as this method does not rely on local sequence features, which are known to vary systematically with chromosomal position [23]. As the only information used for our classification is the presence or absence of orthologs in other genomes, it seems very unlikely that two neighbouring genes’ probabilities to be classified as horizontally transferred are correlated due to methodological biases.

We found that the clustering score for spatial clustering of the more ‘conservative’ and smaller data set (*terminal.pen2*) is, at 2.8, substantially increased compared to the clustering score of the larger set (*terminal.pen1*), at 1.6. This increase is consistent with a true biological relationship between horizontal gene transfer and spatial gene clustering. The *terminal.pen2* dataset is expected to contain less false-positive HGT candidates than *terminal.pen1* (due to the increased gain/loss penalty ratio, stronger support is required before a gene is considered to result from HGT): as the fraction of false positives decreases, the strength of clustering grows.

Our results can be interpreted as strong evidence for horizontal co-transfer, e.g., by the uptake of a complete or partial operon by the host genome. Alternatively, it is conceivable that certain genomic regions (“islands”) may be more suitable for the integration of foreign DNA [8], either due to a mutational bias such as a specific nucleotide composition, or due to a selective bias.

## Conclusions

The observed degree of clustering in general and its dependence on the likely false-positive rate within a candidate set suggests that a certain degree of clustering is a typical feature of reliable candidate sets of recently transferred genes. The spatial clustering is consistent with the horizontal co-transfer of neighbouring genes on a continuous stretch of DNA; the mutational process of HGT does not preferentially transfer individual genes [11], and thus co-transfer is likely if the transferred piece of DNA is large enough. The enrichment of functionally related gene pairs in HGT candidate sets supports the role of HGT in bacterial adaptation; however, because functionally related genes often reside in the same operon, functional and spatial clustering may not be fully independent.

The observed patterns suggest that a quantification of (genomic or functional) clustering may be used as a quality measure for methods that aim to identify horizontally transferred genes: candidate sets produced by different methods (or parameter settings) can be tested against each other. This benchmarking approach may be most useful for methods that detect relatively recent transfers. Furthermore, its application requires that the expected age distributions of HGT events are similar between compared methods, as the strength of clustering likely decreases with increasing age.

Note that this method is only suited to compare false discovery rates, but does not inform about the sensitivity; a dataset constructed using more stringent parameters will likely have fewer false positive predictions of HGT, but may also have more false negatives. For a meaningful comparison of two methods, one should thus also take the absolute numbers of HGT predictions into account. All HGT detection methods that involve tuneable parameters are expected to show a trade-off between sensitivity and specificity, resulting in a negative correlation between size and positive predictive value of HGT candidate sets, just as observed between our *terminal.pen1* and *terminal.pen2* datasets. This phenomenon might be used to select appropriate parameters for a given study.

## Methods

### Identification of horizontally transferred genes

We employ the HGT data described in [22]. Briefly, this data was derived as follows: stringent orthologous single-gene families were identified for 31 γ-proteobacterial strains, based on reciprocal best Blastp-hits (E-value cutoff 10^-40^) among all pairs within one orthologous family. The stringency of orthology assignment can lead to false negative predictions (i.e., a gene is considered absent despite being present in the genome). However, any inaccuracies due to false negatives should only add noise to the analysis, but are not expected to bias the results towards the observed signals. A phylogenetic tree representing vertical inheritance was reconstructed from a concatenated alignment of 114 gene families with one representative in each analysed genome. Note that the grouping of the insect symbionts *Wollbachia, Buchnera,* and *Blochmannia* into a single clade may represent an artifact due to similar compositional biases resulting from their similar lifestyle [24]; however, as we restrict our analyses to the terminal branches, inaccuracies in the phylogeny are not likely to severely bias our results. Because 7 species were classified as outgroups and 3 species had unresolved positions in the tree, horizontally transferred genes were identified for 21 closely related γ-proteobacterial species (or strains of species) on this tree: *E. coli* (4x), *Shigella* (2x), *Salmonella* (3x), *Yersinia* (3x), *Photorhabdus*, *Wigglesworthia*, *Blochmannia*, *Buchnera* (3x), *Haemophilus* (2x), and *Pasteurella*. For each gene family, the presence/absence pattern was projected onto the phylogenetic tree. The most parsimonious explanation for the phyletic pattern in terms of gene gain and loss events across the tree was identified using generalised parsimony (under the DELTRAN model; for details see [22] and [4]).

For the present analysis, we only considered HGT events in the terminal branches of the phylogenetic tree. Thus, a gene is only labelled as horizontally transferred if the most parsimonious scenario includes a gain event after the host genome’s divergence from the last common ancestor with any other genome in our dataset. This approach is conservative and will, compared to including more ancient events, produce less false positive HGT candidates. All other genes were labelled as not horizontally transferred. ‘Singleton’ genes, for which we could not identify any orthologs within our dataset, are prone to be mis-annotations; to be conservative, we also did not label these as horizontally transferred. We further excluded plasmids from the analysis.

If we assume that average genome sizes are roughly constant over evolutionary time, then gene losses and gene gains by horizontal transfer have to balance [25]; this corresponds to a gain/loss penalty ratio *P*_*g*_*/P*_*l*_ = 1 in the parsimony calculation [22]. Conversely, previous authors have argued for a gain penalty that is twice the penalty for losses (*P*_*g*_*/P*_*l*_ = 2) [26]; while this seems appealing due to the much higher mutational probability of gene losses compared to transfers, it leads to an unrealistic decrease in average genome sizes over evolutionary time [25]. Here, we apply both penalty ratios, leading to two candidate sets of horizontally transferred genes. Within 2977 gene families that include one *E. coli* K12 gene plus at least one additional gene in another genome, we identified a total of 3620 horizontal transfers with gain/loss penalty ratio 1 (*terminal.pen1*), and 2272 transfers with gain/loss penalty ratio 2 (*terminal.pen2*). We restricted our analysis to the 2020 (*terminal.pen1*) and 961 (*terminal.pen2*) HGT events mapped to the terminal branches of the phylogeny.

### Statistical model for spatial clustering

Let genome *G* contain *n* genes, out of which a number *c* are HGT candidates (in the candidate set at hand). Set *H*_*i*_ = 1 if the *i*-th gene is considered to be horizontally transferred in the current candidate set. Set *C*_*i*_ = 1 if (*H*_*i+1*_ = 1 or *H*_*i+2*_ = 1 or *H*_*i+3*_ = 1), i.e., if at least one of its three nearest right-hand side neighbours is also transferred; else set *C*_*i*_ = 0. Then count the number of such transferred neighbour pairs:

$$ Coun{t}_G={\displaystyle \sum_{i=1}^n{C}_i.} $$

By including not only immediate genomic neighbours, we partly account for the effect of gene rearrangements after a transfer. By considering up to four genes at the same time, we remain within the length of ~90 % of operons in *E. coli* as estimated based on RegulonDB [27].

The expected value of *Count*_*G*_ under the null model of no spatial association between horizontally transferred genes can be specified algebraically:

$$ E\left( Coun{t}_G\right)=\left(n-1\right)\ast \frac{c}{n}\ast \frac{c-1}{n-1}+\left(n-2\right)\ast \frac{c}{n}\ast \frac{n-c}{n-1}\ast \frac{c-1}{n-2}+\left(n-3\right)\ast \frac{c}{n}\ast \frac{n-c}{n-1}\ast \frac{n-c-1}{n-2}\ast \frac{c-1}{n-3} $$

### Statistical model for metabolic clustering

For *E. coli* K12, detailed and reliable information about functional relationships within the metabolic network is available. Thus, metabolic clustering was only assessed for this strain. We obtained a list of metabolically coupled gene pairs (L_1_) from [28]. Two genes were considered to be coupled if any given flux through the reaction catalysed by one gene enforces a proportional flux through the reaction catalysed by the other (‘full coupling’ in the language of [28]). Thus, at least in *E. coli* K12, the two genes are fully dependent on each other in their function.

For *n* = 4242 examined genes in the *E. coli K12* genome, there are *n* x (*n*-1)/2 possible pairs in total. With L_1_ = 701 identified fully coupled pairs, the *a priori* probability of any given pair to be coupled was thus p_1_ = L_1_/(*n* x (*n*-1)/2). Now consider an HGT candidate set *C* of *n*_*2*_ genes, among which (n_2_ x (n_2_-1)/2) interactions are possible. If metabolic coupling was unrelated to horizontal transfers, we should then expect to find p_1_ x (n_2_ x (n_2_-1)/2) metabolically coupled pairs of horizontally transferred genes.

### Clustering scores and *p* values

To quantify deviations from our null models of no spatial (or no metabolic) clustering, we defined the clustering score *CC* as the observed number of neighbouring pairs divided by the corresponding expected value. *CC* > > 1 indicates strong clustering, while *CC* < 1 indicates that there is less spatial or metabolic clustering than expected.

To assess the significance of our observations, *p* values were computed by randomizations. For ‘spatial’ *p* values, we randomly distributed the observed number of HGT candidates over each of the 31 examined genomes and counted the number of resulting pairs. In our simulations, we assume all examined genes (i.e., potential HGT candidates) to be arranged in a consecutive way in their host genomes, in particular without gaps between them (this corresponds to the statistical model we use for computing the expected value of *Count*_*G*_). However, as described above, we treat all ‘singleton’ genes as non-HGT. This effectively introduces gaps between the examined genes when counting the actual number of observed pairs, which we don’t account for in our statistical model. Thus, the assumed expected values are biased upwards, and the computed clustering score *CC* and the *p* values are conservative.

‘Metabolic’ *p* values were computed in a similar manner, by randomly assigning HGT status to the genes that form the *E. coli K 12* interaction network. The topology of the interaction graph was treated as fixed.

## Reviewers’ comments

### Reviewer’s report 1: Johann Peter Gogarten in collaboration with Luiz Thiberio Rangel, University of Connecticut

The manuscript presents interesting results, namely that recently transferred genes are frequently located near other recently transferred genes, and that these neighboring genes show more frequent metabolic interactions than expected by chance. The methods for the assembly of gene families and HGT inference are consistent. I agree with the authors’ interpretation that this observation is likely caused by the simultaneous transfer and integration of neighboring genes into a recipient genome, although the authors did not provided arguments to refute the alternative hypothesis cited in the last paragraph of the Results and Discussion section (spatial clustering due to integration hotspots). It might be useful to discuss the alternative explanation for transferred genes frequently neighboring each other (integration hotspots) more prominently.

Authors’ response: *We followed this suggestion and now discuss the alternative hypothesis in the background section, when we explain the motivation for our study.*

My main concern is that this manuscript does not do justice to the work others have done regarding the co-transfer of genes:

Phylogenetic profiling [1] is an approach to identify the function of genes due to their frequent co-occurrence in genomes. A whole branch of bioinformatics has emerged implementing better approaches to phylogenetic profiling to infer gene functions and interactions -- the popularity of this approach is illustrated by the foundational publication being cited over 1700 times.

Authors’ response: *now discussed briefly at the end of the**Background**section.*

The relationship between gene clustering and gene transfer was the subject of the selfish operon theory [2]. While this theory had its critiques, I think this theory should at least briefly be summarized, and certainly a proper citation to this work should be included - it does not seem sufficient to cite one paper discussing a possible shortcoming of this theory (the critique in [3] is based on the observation that essential genes are also clustered, from which I would conclude that essential genes are also transferred - for which ample evidence has accumulated [4, 5]).

Authors’ response: *done.*

*Other criticisms:*

Many of the citations in the text are field codes [4] that are not included in the bibliography (e.g., McDaniel 2010, Martin McInerny 2010, ….). In addition, the author date format is used for Chan et al. 2009, but the references are not given in alphabetical order.

Authors’ response: *corrected.*

In the Background section the authors discuss the frequency of **“recent”** gene transfer. The cited manuscript [6] identifies genes acquired in E. coli since the divergence from Salmonella. Many of these transfers are much more ancient that the transfers discussed in this manuscript. Also, **the approach in** [6] **is based on compositional analyses** and will detect only a fraction of the recent transfers between close relatives, whereas the authors’ approach is based on gene presence absence data.

Authors’ response: *we removed “recent”; this section only aims to emphasize the importance of HGT for bacterial evolution.*

Page 4: The discussion of successful transfer describes fixation in the population as one integral step of a successful transfer, and furthermore claims that drift could not be responsible for fixation due to the large population size of “free living bacteria”.

A) A larger bacterial population will likely experience more neutral and nearly neutral transfers into members of the population. If the number of genes acquired by members of the population is proportional to population size, then similar to the rate for neutral substitutions being independent of population size, one should expect that the fixation of neutral acquired genes due to drift is independent of populations size. The impact of population size is on what is seen by selection as advantageous or detrimental, i.e. a large population will have fewer nearly neutral acquired genes. Many recently acquired genes in individual bacterial genomes appear to not increase the fitness of the recipient, and many of these genes are likely to be lost from the population on the long run [4, 7].

Authors’ response: *Some neutral sequences may indeed be fixed by drift alone. However, there appears to be a deletion bias in bacterial genomes* [15]*; sequences that have no beneficial fitness effect are thus unlikely to survive for long periods. This is now discussed fully.*

B) many of the strains included in this study are not free-living and undergo frequent bottlenecks.

Authors’ response: *we rewrote this section and removed the adjective “free-living”.*

C) The presented analysis does not distinguish between genes being fixed in a population and genes being present in only a few members of the population that were cultured and sequences.

Authors’ response: *This is of course true, as we only analyzed individual genomes; this is now discussed.*

D) The strong black queen hypothesis suggests that genes may be useful for a population without being fixed in all members of the population [8, 9].

Authors’ response: *The fixation of all observed horizontally transferred genes is not essential to our argument, which is now spelt out in the**Background**section.*

Page 4, line 13: No citation is provided for the assumption that if two genes are transferred together, they will likely be fixed together. One solution would be to turn this into subjunctive mood (e.g. “they may be integrated into the host genome together.”

Authors’ response: *changed as suggested.*

Page 4, line 42: Citation of [10] would be appropriate here.

Authors’ response: *indeed; done.*

Page 5, line 27: “genomic distance” should be defined as distance of genes within a genome. At first reading many will associate the term with distance between genomes.

Authors’ response: *clarified as suggested.*

Page 6, line 47: HGT consists of two processes, both of these can create a bias. Hotspots for gene integration could lead to clustering of genes integrated into the genome. The genomic uptake sequences would bias what is taken into the cell, but I am not aware that these will impact the region where sequences are integrated.

Authors’ response: *clarified.*

Page 8 line 55: These are not different species as claimed. Many of these are strains belonging to the same species. See the above comment on fixation.

Authors’ response: *we replaced “species” by “genomes” or “strains” where appropriate.*

In describing the HGT mechanisms the authors could mention “nanotubes” [11]

Authors’ response: *done.*

The authors only use only transfers into the terminal branches in this study, thus I don’t think that the uncertainly of the assumed genome phylogeny (which is taken from [12]) matters much, but I think it worthwhile to point out that the grouping of the insect symbionts, Wollbachia, Buchnera and Blochmannia (the authors consistently use a different spelling, but Friedrich Blochmann spelled his name with two “n”) into a single clade may represent an artifact due to similar compositional bias resulting from their similar lifestyle [13].

Authors’ response: *now discussed.*

It would be interesting to indicate the standard deviation expected from the random distributions used as null hypothesis. Also, are the distributions parameterized to calculate the P-value? If yes, which distribution is assumed?

Authors’ response: *The distributions are not parameterized; thepvalues are calculated simply as (number of randomizedCC* ≥ *CC*_observed_ *+ 1)/(number of randomizations +1), where the “+1” terms account for the observation itself.*

### References used by Reviewer 1

1. Pellegrini M, Marcotte EM, Thompson MJ, Eisenberg D, Yeates TO: Assigning protein functions by comparative genome analysis: protein phylogenetic profiles. Proc Natl Acad Sci U A 1999, 96:4285–8.

2. Lawrence JG, Roth JR: Selfish operons: horizontal transfer may drive the evolution of gene clusters. Genetics 1996, 143:1843–1860.

3. Pál C, Hurst LD: Evidence against the selfish operon theory. Trends Genet TIG 2004, 20:232–234.

4. Gogarten JP, Townsend JP: Horizontal gene transfer, genome innovation and evolution. Nat Rev Microbiol 2005, 3:679–87.

5. Andam CP, Gogarten JP: Biased gene transfer in microbial evolution. Nat Rev Microbiol 2011, 9:543–55.

6. Lawrence JG, Ochman H: Molecular archaeology of the Escherichia coli genome. Proc Natl Acad Sci USA 1998, 95:9413–9417.

7. Lawrence JG: Selfish operons and speciation by gene transfer. Trends Microbiol 1997, 5:355–359.

8. Fullmer MS, Soucy SM, Gogarten JP: The pan-genome as a shared genomic resource: mutual cheating, cooperation and the black queen hypothesis. Front Microbiol 2015, 6:728.

9. Morris JJ, Lenski RE, Zinser ER: The Black Queen Hypothesis: evolution of dependencies through adaptive gene loss. mBio 2012, 3.

10. Jain R, Rivera MC, Lake JA: Horizontal gene transfer among genomes: the complexity hypothesis. Proc Natl Acad Sci U A 1999, 96:3801–6.

11. Dubey GP, Ben-Yehuda S: Intercellular nanotubes mediate bacterial communication. Cell 2011, 144:590–600.

12. Lercher MJ, Pál C: Integration of horizontally transferred genes into regulatory interaction networks takes many million years. Mol Biol Evol 2008, 25:559–567.

13. Herbeck JT, Degnan PH, Wernegreen JJ: Nonhomogeneous model of sequence evolution indicates independent origins of primary endosymbionts within the enterobacteriales (gamma-Proteobacteria). Mol Biol Evol 2005, 22:520–532.

### Reviewer’s report 2: Yuri Wolf, NCBI

Dilthey and Lercher find that genes, predicted to be recent acquisitions in individual gamma-proteobacteria, are non-randomly distributed in the recipient genomes. The topic is quite interesting and the general approach is reasonable. At the same time the specific methods, as implemented by the authors, are a bit too coarse in my opinion.

### Reviewer recommendations to authors

First, the “stringent orthologous single-gene families”, identified “based on reciprocal best Blastp-hits (E-value cutoff 10^-40) among all pairs within one orthologous family” (Methods) are prone to false negatives due to the very stringency of the criteria. False negatives manifest themselves as gaps in the presence/absence pattern and, in the course of the analysis, can lead to falsely predicted acquisitions. Full-scale construction of orthologous families is prohibitively expensive if attempted for many thousands of completely sequenced microbial genomes, available today, but for the set of 31 gamma-proteobacteria it is a relatively trivial exercise in bioinformatics.

Authors’ response: *we agree that alternative methods with fewer false positives exist. However, any inaccuracies due to false negatives should only add noise to the analysis, but are not expected to bias the results towards the observed signal. This is now stated in the manuscript.*

Second, along the same lines, parsimony analysis is somewhat too blunt a tool to infer gene acquisitions. At the given scale there is absolutely no reason not to use probabilistic methods of inference that are easily available (like EREM, COUNT or GLOOME; a large family of Bayesian inference methods can also be adapted to the task). The primary advantage of these estimates is that they are not dependent on the gain/loss ratio, declared *a priori*, but infer it from the data. Also, most such methods provide posterior probabilities to the inferred events, which could serve as a more natural stringency parameter.

Authors’ response: *probabilistic methods assume a constant rate of gene acquisition (and loss) along the phylogeny. If we assume that HGT occurs in bursts in response to environmental changes (which seems likely, see, e.g., Pal et al. 2005), then this assumption is not warranted. Thus, it seems far from clear that probabilistic methods outperform parsimony unless they are informed about environmental changes.*

Finally, the assumption of the random model, to which the authors compare the empirical data, are somewhat unrealistic. It is too much to expect that a newly acquired gene would be inserted into the recipient genome anywhere with equal probability. One can argue that the very existence of locations, effectively prohibited as insertion sites (e.g. right in the middle of F0 ATPase operon), is sufficient to reject the postulated random model even if the all acquired genes are acquired independently. I would suggest to use the opposite extreme - to allow random insertions only between predicted directons - in the test. If even this model is incompatible with the data, the case for concerted acquisitions would be much stronger. To forestall an obvious argument “yes, these are rough estimates, but they should be good enough as a first approximation”, I would say that probably there is no doubt in the community that gene acquisitions are not distributed strictly randomly. It is the quantitative component that is of much interest (what fraction of acquisitions those concerted events comprise), and the methods, chosen by the authors, make these quantitative estimates less reliable.

Authors’ response: *we indeed chose the simplest conceivable null model, which will not be a faithful representation of HGT insertion positions even if all horizontally transferred genes indeed integrated independently. The alternative model suggested by the reviewer appears overly conservative, but would certainly help to better quantify the amount of clustering. However, we see the main contribution of this paper in its suggestion of a benchmark for HGT detection methods (see also below), and for this the chosen model appears sufficient.*

On the technical/presentation side, there are many small glitches that need to be fixed in the final version. There are **multiple problems with reference formatting**, like “gene transfer agents [8, 9]” (p. 4 and throughout the whole text).

Authors’ response: *corrected.*

I also have a general technical suggestion: as the authors perform permutation analysis to estimate the p-values (p. 10), the analytically computed expectations (p. 9) are, essentially, unnecessary. The advantage of using simulations-derived expectations is in that it would allow analysis under more sophisticated random models (e.g. with explicitly defined gene insertion hotspots) that might be easily implemented directly, but difficult for analytical solution.

Authors’ response: *agreed. We do however prefer to provide an analytical expectation where this is possible.*

## Abbreviation

HGT: horizontal gene transfer
